# Deep water pathways in the North Pacific Ocean revealed by Lagrangian particle tracking

**DOI:** 10.1038/s41598-022-10080-8

**Published:** 2022-04-22

**Authors:** T. Kawasaki, Y. Matsumura, H. Hasumi

**Affiliations:** Atmosphere and Ocean Research Institute, The University of Tokyo, Chiba, Japan

**Keywords:** Ocean sciences, Physical oceanography

## Abstract

Lagrangian particle tracking experiments are conducted to investigate the pathways of deep water in the North Pacific Ocean. The flow field is taken from a state-of-the-art deep circulation simulation. An unprecedented number of particles are tracked to quantify the volume transport and residence time. Half of the North Pacific deep water returns to the Southern Ocean, and its principal pathway is along the western boundary current in the Southwest Pacific Basin in the deep layer. About 30% is exported to the Indian Ocean after upwelling to the shallow layer in the western North Pacific Ocean. The rest is transported to the Arctic Ocean through the Bering Strait or evaporates within the Pacific Ocean. Upwelling of deep water is confined in the western North Pacific Ocean owing to the strong vertical mixing. The mean residence time of deep water in the North Pacific Ocean is estimated to be several hundred years, which is consistent with the observed radiocarbon distribution.

## Introduction

The North Pacific Ocean is known as the endpoint of the global thermohaline circulation which starts from the deep water formation in the northern North Atlantic Ocean and around Antarctica^1–3^. Because of the lack of deep water formation, temperature and salinity in the deep North Pacific Ocean are distributed uniformly in horizontal relative to the other basins, making it difficult to describe the pathway and volume transport of deep water from observed temperature and salinity distributions. Indirectly estimating transport from the observed distribution of temperature and salinity, some studies suggested that a significant amount of water enters the North Pacific Ocean in the bottom layer (8 Sv at 10°N by Wijffels et al.^4^; 5 Sv at 24°N by Lumpkin and Speer^5^; 1 Sv = 10^6^ m^3^ s^-1^) while another study^3^ suggested that little deep water enters to the north of 24°N. There neither is a consensus on the fate of the deep water in the North Pacific Ocean, i.e., towcard which basin the upwelled water flows, among such studies.

Direct measurements of deep currents are of use in estimating the transport in the bottom layer of the North Pacific Ocean and tracing its origin as the topography constrains the water to flow through narrow passages and concentrated mooring of current meters is feasible there^6–8^. Geostrophic calculation based on densely sampled temperature and salinity across narrow passages also gives precise estimates of transport. By integrating existing estimates based on such methods, Kawabe and Fujio^9^ described the transport and pathway of deep water in the North Pacific Ocean below 3500 m in detail. However, less is known for the above 3500 m from observations.

Lagrangian particle tracking is a useful method for analyzing the ocean circulation field obtained by numerical ocean models. It is particularly suitable for revealing the route and travel time of water^10^. Although this method is being extensively applied, only a few studies have investigated the deep circulation of the North Pacific Ocean so far. Shah et al.^11^ utilized the flow field obtained by an ocean circulation inverse model with 2° horizontal resolution and 24 vertical levels and tracked the particles initially placed uniformly in the deep North Pacific Ocean. Although they described routes and travel times for selected particles, they did not statistically discuss the pathways nor quantitatively estimate the circulation. The flow field calculation adopted 0.1 × 10^–4^ m^2^ s^-1^ for vertical diffusivity uniformly, which is too small compared with observation- and model-based estimates^12,13^. The tide-induced vertical mixing, which induces the buoyancy gain of deep water, is the main mechanism to maintain the upwelling of deep water^14,15^. Such small vertical diffusivity leads to considerable underestimation of the deep circulation in the North Pacific Ocean^16,17^.

Here, we investigate the pathway of deep water in the North Pacific Ocean by tracking Lagrangian particles. By endowing particles with the property of volumetric transport, we try to quantitatively describe the three-dimensional structure of the North Pacific Ocean circulation. The flow field is taken from the OGCM calculation by Kawasaki et al.^18^, which incorporated the latest findings on vertical diffusivity and reasonably reproduced the distribution of radiocarbon^19^, an indicator for the age of water. For comparison purpose, another flow field simulated under an empirical distribution of the vertical diffusivity is also utilized. The former case is called the “case 3D”, and the latter the “case 1D” (see “Methods”). Unless otherwise stated, the results of the case 3D are described.

## Results

### Volume transport and residence time

**Figure 1:**
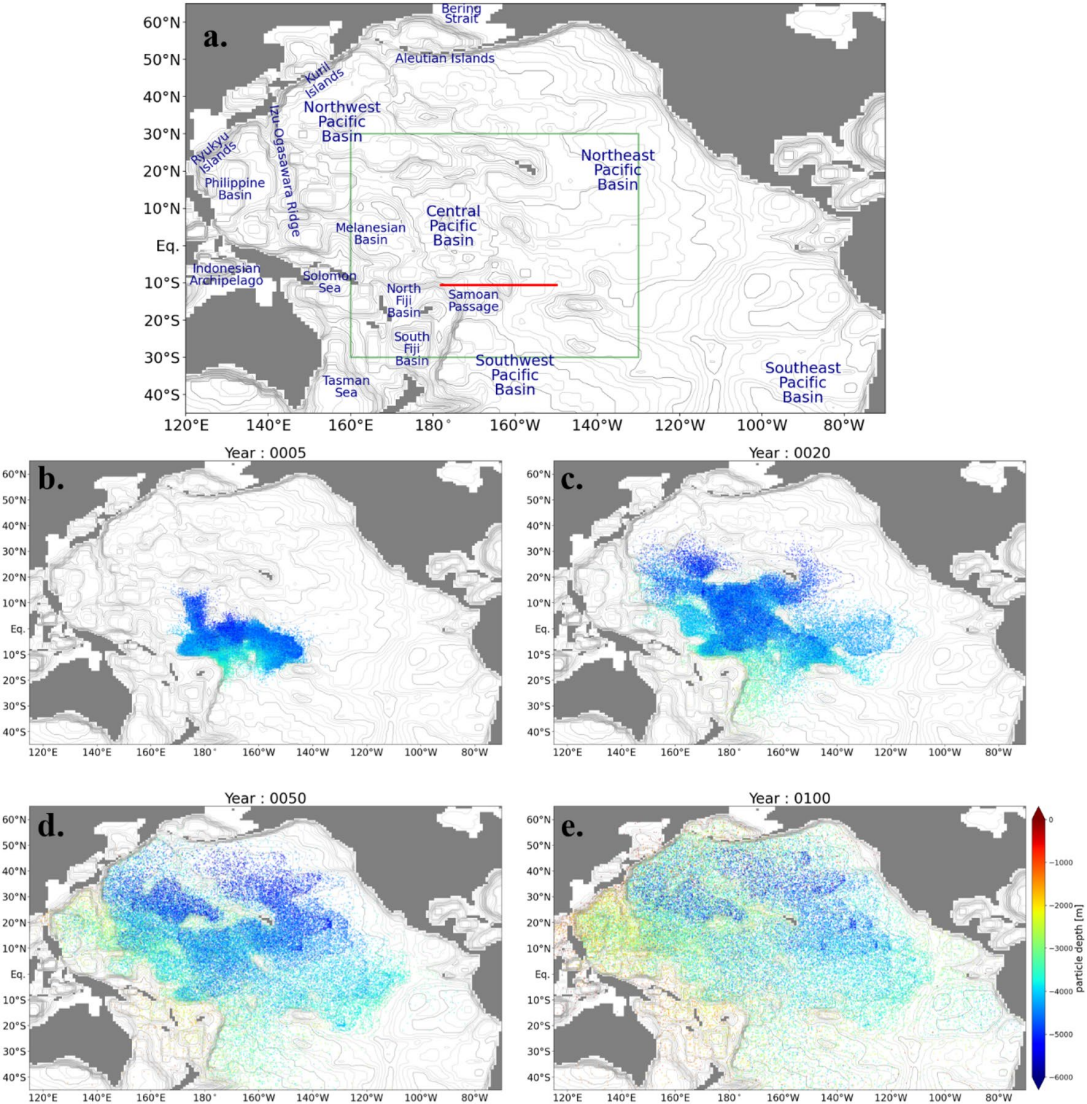
(**a**) Map of the Pacific Ocean. The red line indicates the section where the Lagrangian particles are released. The green box indicates the range of Fig. 7b. Positions of the Lagrangian particles (**b**) 5 years, (**c**) 20 years, (**d**) 50 years, and (**e**) 100 years after particle tracking started. The color indicates the depth of particles. For readability, one in every 47 particles is plotted. The gray contour indicates the seafloor depth in our model. The thick and thin contour intervals are 200 and 1000 m, respectively.

The particles are released from the Samoan Passage and its adjacent pathways below 3500 m depth (Fig. 1a). To illustrate how the particles move, the positions of the particles at several times are shown in Fig. 1b–e. The movie of particles is also shown in the supplemental material (Movie S1). Some of the particles immediately rise to the deep layer (2000–3500 m depth) and return to the Southern Ocean through the Southwest Pacific Basin (Fig. 1b and c). The rest of the particles are transported to the North Pacific basins (the Central, Northwest, and Northeast Pacific Basins) by the horizontal currents in the bottom layer which are constrained by the bottom topography (Fig. 1c and d). Then, these particles rise to the deep layer mainly in the western North Pacific Ocean (Fig. 1e). They eventually ascend to the intermediate and shallow layers (< 1000 m depth) and reach the Indian and Arctic Oceans through the Indonesian Archipelago and the Bering Strait, respectively (Movie S1).

**Figure 2:**
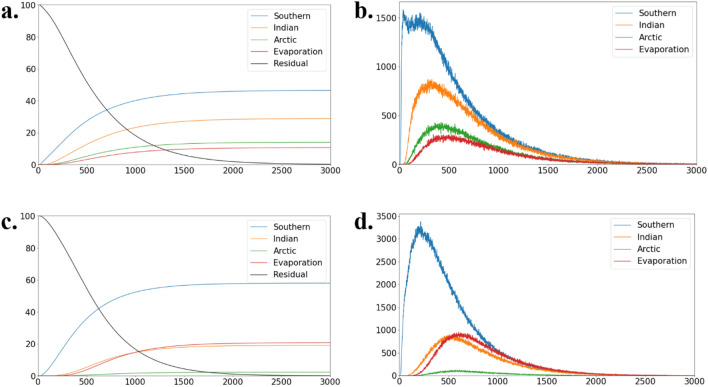
(**a**) The cumulative ratio (%) of the number of particles reaching each basin. (**b**) The number of particles reaching each basin in each year. (**c, d**) same as (**a, b**), but in the case 1D.

Almost all (~ 99.8%) of the particles leave from the Pacific Ocean or evaporate by 3000 years later (Fig. 2a). Since the number of particles is proportional to volume in this method, the transport is calculated by counting the particles reaching other basins. 3.50, 2.17, and 1.05 Sv (46.5, 28.8, and 13.9%) of bottom water originated from the Samoan Passage (and its adjacent passages) is transported to the Southern, Indian, and Arctic Oceans, respectively, and 0.80 Sv (10.3%) evaporates within the Pacific Ocean (Table 1). Several observation-based studies^2,5,9^ assumed that all (~ 10 Sv) of the Pacific deep water returns to the Southern Ocean in the deep layer. On the other hand, Talley^3^ estimated about 9, 4, and 1 Sv of the Pacific deep water originated from the Southern Ocean are exported to the Southern, Indian, and Arctic Oceans, respectively. Our result is qualitatively consistent with this estimate^3^ although the modeled export to the Southern and Indian Oceans is smaller. The modeled export to the Arctic Ocean is a bit larger than the observational estimate^20^ of the net volume transport through the Bering Strait (~ 0.8 Sv).

**Table 1:** Number and ratio of particles, volume transport, and residence time of particles for each destination. The mean, median, and mode are listed for the residence time. The result is obtained from the tracking for 3000 years. The total number of particles is 2,342,025.

Destination	Number of particles	Ratio of particle (%)	Volume transport (Sv)	Residence time (year)		
				Mean	Median	Mode
Southern Ocean	1,088,079	46.5	3.50	533	409	40
Indian Ocean	675,128	28.8	2.17	672	559	318
Arctic Ocean	326,424	13.9	1.05	728	620	421
Atmosphere	248,321	10.6	0.80	804	696	501
Residual	4,073	0.2	0.01	–	–	–

The residence time (seawater age) of the deep Pacific water is equivalent to the time required for particles to reach other basins. Its statistics are summarized in Table 1. The mean required time to reach the Southern, Indian, and Arctic Oceans and the atmosphere (evaporation) is 533, 627, 728, and 804 years, respectively. The median time is shorter than the mean time by about 100 years for each destination, and the mode time is even shorter because of the right-skewed frequency distribution in the arrival time (Fig. 2b). Note that the “mode time” means the year when the number of particles reaching each destination is largest. The residence time of particles is less than 1000 years and is consistent with an observation-based estimate of water age in the deep Pacific Ocean^21^.

### Main pathway

**Figure 3:**
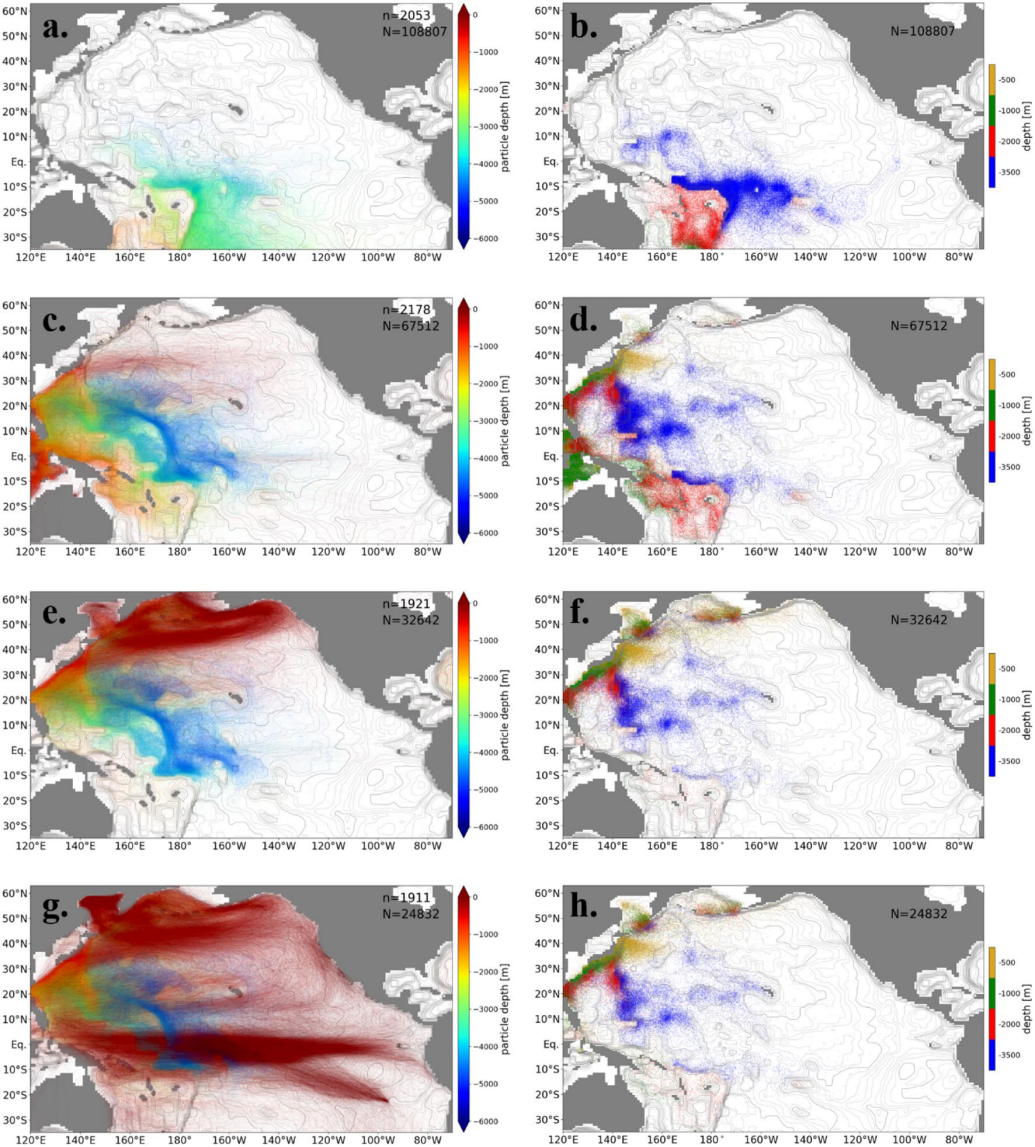
The pathways defined by the earliest 10% of the particles that leave from the Pacific Ocean to each destination. Left-hand panels show trajectories of the particles. Colors indicate depths. The numbers of total particles (N) and plotted particles (n) are written in the panels. The right-hand panels show the horizontal positions where particles first cross the 500 (yellow), 1000 (green), 2000 (red), and 3500 m depths (blue). The destinations are (**a, b**) the Southern Ocean, (**c, d**) the Indian Ocean, (**e, f**) the Arctic Ocean, and (**g, h**) the atmosphere (evaporation).

**Figure 4:**
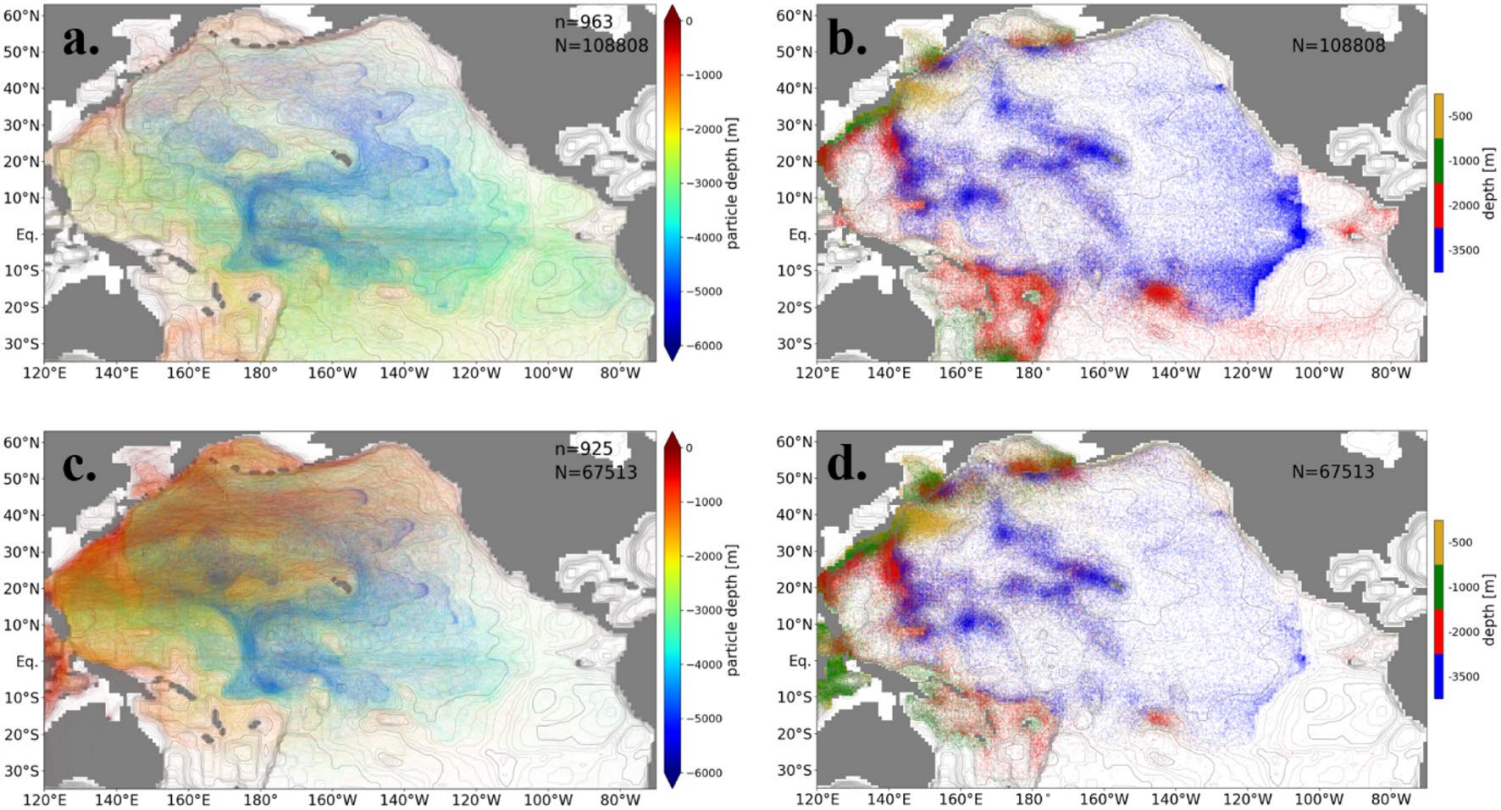
Same as Fig. 3, but the particles with around the median residence time (45–55%) in the Pacific Ocean. The particles reaching the Arctic Ocean and atmosphere (evaporation) are not shown.

The main pathways, defined in terms of particle tracking, are the routes where a large number of particles pass through. Therefore, the main pathways are visualized by superimposing the trajectories of the particles (left panels in Figs. 3 and 4). To identify the shortest pathways, the trajectories of the earliest 10% of the particles that leave the Pacific Ocean are extracted for each destination together with the horizontal positions where these particles first cross the depths dividing the bottom, deep, intermediate, and shallow layers (“ascending points” hereafter) (Fig. 3).

Most of the particles reaching the Southern Ocean ascend near the Samoan Passage (e.g., Melanesian Basin) and are transported southward in the Southwest Pacific Basin without crossing the equator (Fig. 3a and b). Some particles ascend in the Melanesian Basin or Southwest Pacific Basin and reach the Southern Ocean through the South Fiji Basin and the Tasman Sea (Fig. 3a and b), which corresponds to the Tasman leakage^22^.

Except for the shallow layer (< 500 m depth), a majority of particles reaching the Indian Ocean through the Indonesian Archipelago are transported in the western North Pacific Ocean, not in the east (Fig. 3c). The particles are transported in the Central Pacific Basin or the Melanesian Basin in the bottom layer, then join and ascend to the deep layer at the Izu-Ogasawara Ridge where turbulent vertical mixing is strong (Fig. 3c and d). These particles ascend to the intermediate layer (500–1000 m depth) at the Luzon Strait (around 20°N, 120°E) and around the Ryukyu Islands and are transported to the Indonesian Archipelago (Fig. 3c and d). The particles on the secondary pathway ascend to the deep layer in the Melanesian Basin and to the intermediate layer in the Solomon Sea or North/South Fiji Basin and reach the Indonesian Archipelago (Fig. 3c and d).

The trajectories and ascending points of most of the particles reaching the Arctic Ocean are similar to those reaching the Indian Ocean in the deep and bottom layers (Fig. 3e and f). In the shallow and intermediate layers, the particles are transported by the Kuroshio, its extension, and the North Pacific subarctic gyre, and reach the Bering Strait (Fig. 3e). The particles on the secondary pathway ascend around the Kuril and Aleutian Islands, where the turbulent vertical mixing is locally enhanced, from the bottom layer to the shallow layer (Fig. 3f).

The trajectories and ascending points of particles evaporating within the Pacific Ocean are similar to those reaching the Arctic Ocean below the intermediate layer (> 1000 m depth). Evaporation of particles mainly takes place in the equatorial Pacific Ocean (figure not shown). The particles are transported to the low-latitude region by the North Pacific subtropical gyre and the Equatorial Counter Current in the shallow layer (Fig. 3g and h).

The particles which reside longer in the North Pacific Ocean follow different pathways. Here, the trajectories are extracted for the particles with around the median residence time (between 45 and 55 percentiles) in the Pacific Ocean. (Fig. 4). The returning routes in the deep layer to the Southern Ocean are localized in the western part of the Southwest Pacific Basin for the short-staying particles (Fig. 3a and b), while the routes of the medium-term-staying particles are distributed over the whole Pacific Ocean (Fig. 4a and b). In summary, the main pathway of particles transported to the Southern Ocean in the deep layer is found in the western part of the Southwest Pacific Basin, and the secondary is found in the Southeast Pacific Basin. This main pathway differs from previous suggestions inferred from the distribution of temperature and salinity: along the eastern edge of the Southwest Pacific Basin^2^ or eastern boundary of the Pacific Ocean^9^.

The pathway of the short-staying particles that reach the Indian Ocean is localized in the western Pacific Ocean (Fig. 3c), while the pathway of medium-term staying particles spreads over the Pacific Ocean (Fig. 4c). The ascending points are also distributed in the whole Pacific Ocean in the bottom and deep layers (Fig. 4d). These features of the pathway are similar for the particles reaching the Arctic Ocean and the atmosphere (figure not shown).

Even when the trajectories are randomly selected from all particles, the main pathways are little influenced compared with the case of medium-term staying particles (Figure S1). They are, however, obscured because the slower particles are more likely to be scattered over the whole deep Pacific Ocean.

### Dependency on flow field

**Figure 5:**
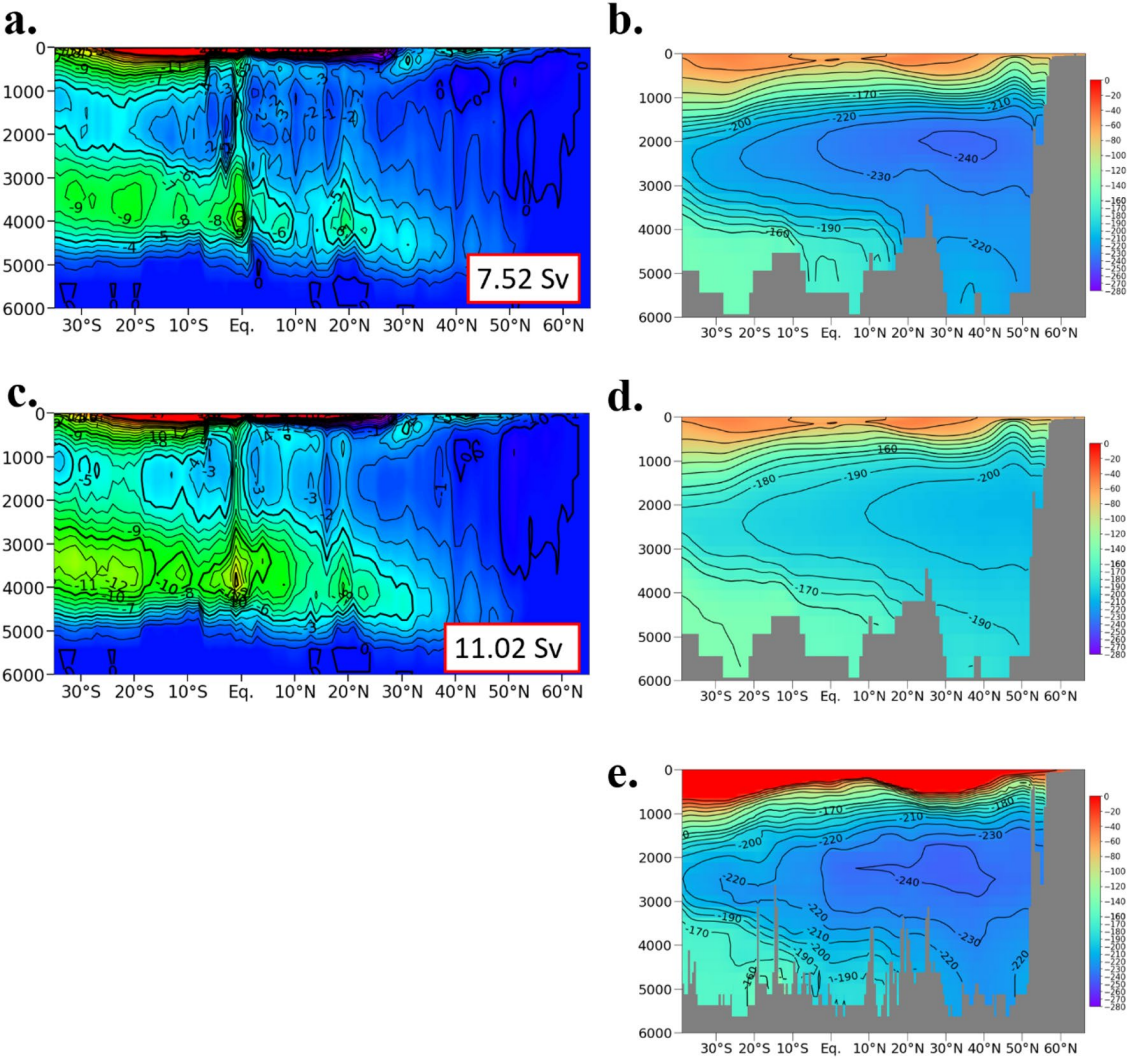
(**a**) Zonally integrated stream function in the Pacific Ocean for the case 3D. Positive for clockwise circulation. The thick and thin contour intervals are 5 and 1 Sv, respectively. The number indicates the northward volume transport at the Samoan Passage and its adjacent pathways. (**b**) Simulated Δ^14^C along 170°W (central Pacific Ocean). The unit is ‰. Contour intervals are 20 and 10 ‰ for larger and smaller than − 160 ‰, respectively. (**c, d**) Same as (**a, b**) but for the case 1D. (**e**) The same as (**b**) but the observed data constructed through the Global Ocean Data Analysis Project Version 2 (GLODAP v2)^19^.

Because the northward transport of bottom water at the Samoan Passage (and its adjacent passages) in the case 1D is larger than that in the case 3D, the number of released particles is also larger in the case 1D (Fig. 5a and c). Almost all the particles leave the Pacific Ocean after 3000 years (Table 2; Fig. 2c). The ratio of the particles reaching the Southern Ocean (57.9%) is larger than that in the case 3D (46.5%), while the ratio of particles reaching the Indian (19.1%) and Arctic Oceans (2.2%) are smaller (Tables 1 and 2). The residence time of the particles reaching the Southern Ocean is smaller than that in the case 3D, while that for the Indian and Arctic Oceans is larger (Tables 1 and 2, and Fig. 2b and d). This behavior of the particles in the case 1D is associated with the small vertical mixing in the deep layer.

**Table 2:** Same as Table 1 but for the case 1D. The total number of particles is 3,475,625.

Destination	Number of particles	Ratio of particle (%)	Volume transport (Sv)	Residence time (year)		
				Mean	Median	Mode
Southern Ocean	2,013,649	57.9	6.47	497	399	219
Indian ocean	662,513	19.1	2.13	772	681	589
Arctic ocean	78,129	2.2	0.25	827	737	571
Atmosphere	719,078	20.7	2.31	855	765	602
Residual	2,256	0.1	0.01	–	–	–

**Figure 6:**
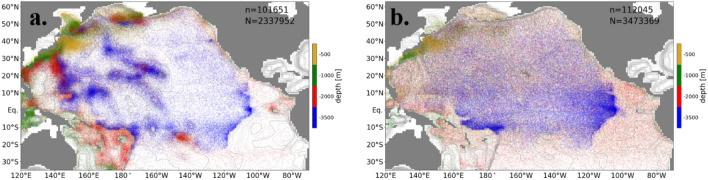
The horizontal positions where particles first cross the 500 (yellow), 1000 (green), 2000 (red), and 3500 m depths (blue) in (**a**) the case 3D and (**b**) the case 1D.

The ascending points of particles are localized around the ridges, seamounts, and boundaries in the case 3D, while they are homogeneously distributed in horizontal in the case 1D as a result of the horizontally uniform vertical diffusivity (Fig. 6). The strong vertical mixing localized in the western North Pacific Ocean in the case 3D causes the efficient ascending of particles and short required time to reach the Indian and Arctic Oceans (Fig. 2d, Tables 1).

## Discussion

The particle tracking demonstrated that about half of deep water in the North Pacific Ocean is exported to the Southern Ocean. Kawabe and Fujio^9^ estimated all of the deep Pacific water is exported to the Southern Ocean in the deep layer, while Talley^3^ estimated about two-thirds of the deep Pacific water is exported to the Southern Ocean. Our result (about half of the deep water returns to the Southern Ocean) is consistent with, but slightly smaller than, Talley’s estimate^3^. The several hundred years of the mean residence time of deep water in the North Pacific Ocean is not consistent with the conventional understanding of the time scale of the deep Pacific Ocean circulation, which was considered to be several thousand years^23,24^, but is consistent with an estimate based on radiocarbon^21^. The principal pathway to the Southern Ocean in the deep layer is along the western boundary of the Southwest Pacific Basin, which is different from what was suggested by Kawabe and Fujio^9^ (along the eastern boundary of the Pacific Ocean). It should be noted that the fate of flow into the North Pacific Ocean in the deep layer, whose existence is suggested by Kawabe and Fujino^9^, is not treated in this study.

An insufficient number of particles may prevent isotropic diffusion representation and bias the pathways. To verify whether the number of particles tracked in this study is sufficient to estimate the volume transport, we divide the particles into 11 groups and performed the same analysis. As a result, the spread of the estimate is within 1% (e.g., 3.482 ~ 3.505 Sv for volume transport to the Southern Ocean in the case 3D). The horizontal diffusivity itself has uncertainty. We conducted the particle tracking with halved or doubled horizontal diffusivity. The mean residence time is longer/shorter by about 20% for the halved/doubled horizontal diffusivity (Table S1). This result suggests the nonnegligible sensitivity of the deep water age to horizontal mixing. The reality of horizontal mixing and its influence on the deep Pacific Ocean circulation is left to be investigated.

Coarse-resolution models, as utilized in this study, cannot adequately represent western boundary currents in the shallow layer (such as the Kuroshio and the Oyashio) and the intermediate layer circulation. Therefore, it is necessary to use a velocity field obtained by an eddy-resolving ocean model to estimate the pathways in the shallow and intermediate layers more accurately. There are several studies applying particle tracking to eddy-resolving models^25,26^, but none for long time scales as in this study. There is difficulty arising from not only the huge size of computation and data to handle but also how to properly consider interannual variability associated with oceanic internal variations. We are currently tackling this problem.

## Methods

### Velocity and diffusivity fields

As the velocity and diffusivity fields for particle tracking, we utilize the results of an ocean general circulation model experiment conducted by the previous study (experiment CTRL of Kawasaki et al.^18^). The horizontal resolution of the model is 1 degree. We applied constant diffusivity of 1000 m^2^ s^-1^ for the horizontal unresolved-eddy-induced mixing. In this experiment, the three-dimensional distribution of vertical turbulent mixing, to which the deep Pacific Ocean circulation is highly sensitive, is given based on the results of a high-resolution tide model^27^ and theoretical and observational studies^28–31^. This circulation field and the particle tracking using this field are named the “case 3D”. For comparison purposes, the velocity and diffusivity fields calculated by prescribing an empirical distribution of vertical diffusivity (Case III of Tsujino et al.^16^), which is horizontally uniform and varies only in vertical, in the same ocean model are also used for particle tracking (case 1D).

Since the employed circulation field does not explicitly resolve mesoscale eddies and further smaller features and is driven by monthly mean sea surface boundary conditions, both high-frequency and interannual variabilities of ocean currents are not simulated. Therefore, the climatological (30-year mean) monthly mean velocity and diffusion fields are utilized for particle tracking. The bolus velocity associated with the thickness diffusion parameterization^32^ applied in the model is added to the velocity field. Note that the bolus velocity is small and does not have a significant impact on particle tracking due to the relatively flat isopycnals in the deep Pacific Ocean.

The northward transport of deep water at the Samoan Passage and its adjacent pathways (~ 10°S) in the bottom layer (> 3500 m depth) is 7.52 and 11.2 Sv (Fig. 5a and c) in the cases 3D and 1D, respectively. Although this is smaller than the estimate from mooring measurements^6,8,33^ (~ 9–10 Sv below 4000 m depth), the simulated radiocarbon distribution is quantitatively and qualitatively consistent with the observation in the deep Pacific Ocean in the case 3D (Fig. 5b and e). Because the radiocarbon is an indicator of how long it has taken since seawater is isolated from contact with the atmosphere, the qualitative and quantitative consistency means the validity of the structure and strength of the deep Pacific Ocean circulation. In the case 1D, the northward volume transport is larger than the observational estimation, and the calculated radiocarbon is also too high, indicating too short residence time of deep water in the North Pacific Ocean (Fig. 5d and e). Note that the northward volume transport at the Wake-Island Passage (18°N, 169°E) and its western passages below 3500 m depth is 3.04 and 3.35 Sv in the cases 3D and 1D, respectively, which are smaller than observed transport (a little more than 3.6 Sv below 3670 m)^7^.

### Particle tracking

The Lagrangian particle tracking system used in the present study is the same as in Nakano et al.^25^, which is a part of an ocean modeling system *kinaco* (source code available via http://lmr.aori.u-tokyo.ac.jp/feog/ymatsu/kinaco.git). The trajectory of each particle is integrated by the fourth-order Runge–Kutta method using the combination of the bilinear-interpolated velocity field (sum of the resolved current and the parameterized bolus velocity) and the turbulent mixing represented by normal-distributed random displacement whose standard deviation corresponds to the unresolved-eddy-induced diffusivity.

**Figure 7:**
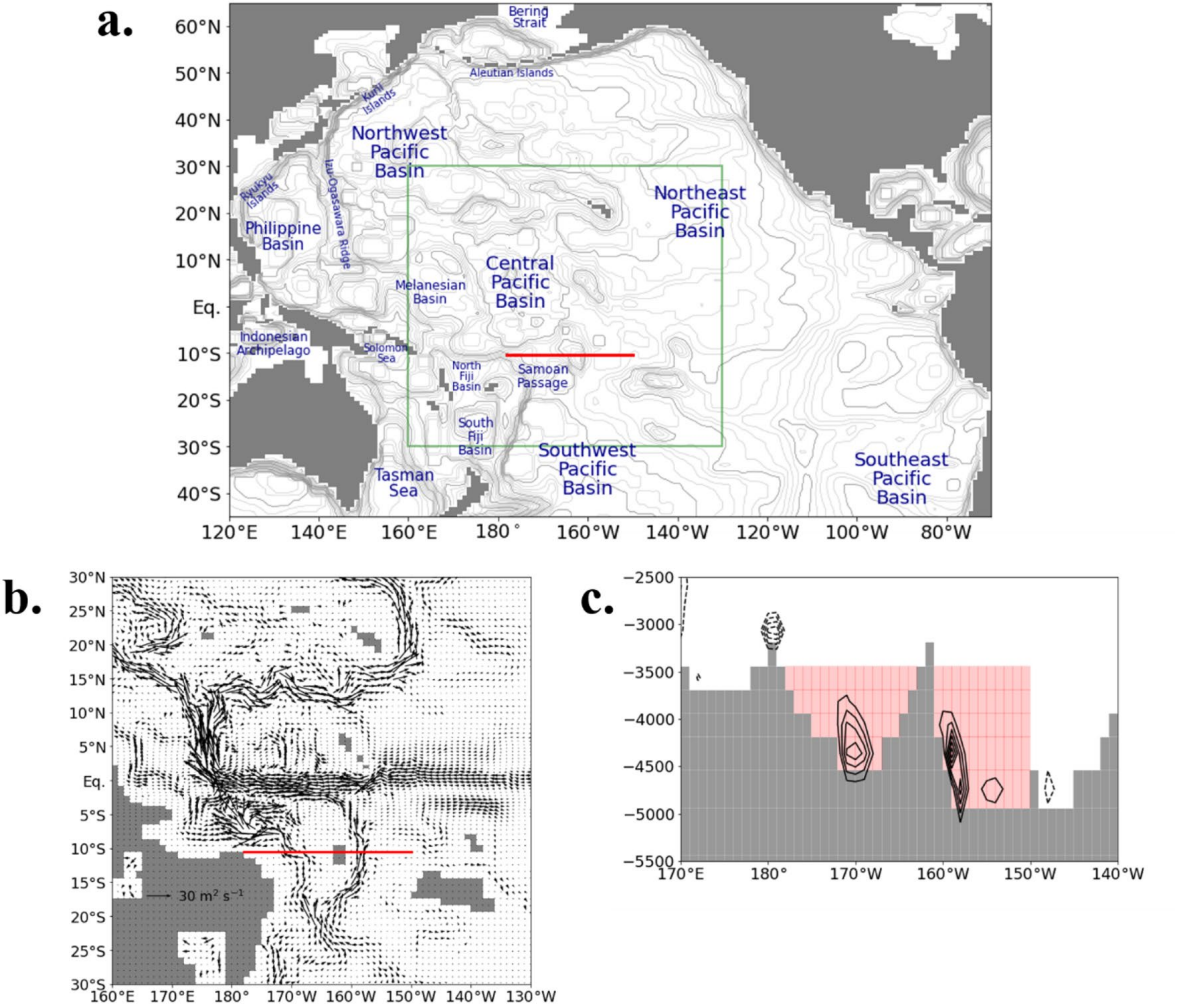
(**a**) Vertically integrated annual-mean horizontal velocity below 3500 m depth. (**b**) Annual-mean meridional velocity along 10°S. The contour interval is 0.005 m s^-1^ (zero line is not drawn). The solid and dotted lines show the northward and southward transport, respectively. The red line and pink shade indicate the section where the Lagrangian particles are released.

Particles are released from the Samoan Passage and its adjacent pathways below 3500 m depth where the bottom water heading to the North Pacific Ocean from the Southern Ocean always passes due to the topographic constraint (Fig. 7). One particle is released every time the northward volume transport reaches 1 × 10^8^ m^3^ on the prescribed section in the passage (shown by a red line in Fig. 7). By so doing, a quantitative evaluation of the pathway is feasible by tracking particles.

Particles are released only for the first year. The total numbers of released particles for the cases 3D and 1D are 2,342,025 and 3,475,625, which correspond to the deep water volume transport of 7.53 and 11.2 Sv, respectively. Each particle stores an index for extinction which is initially zero. It increases by the rate of evaporation at the sea surface when the particle resides in the top-most layer of the model (2 m thick). When the accumulated value for any particle exceeds half of the top-most layer thickness (1 m), the particle is removed by considering the volume of water represented by the particle has evaporated out.

By tracking these particles, it is possible to determine the pathways of the deep water that has passed through the Samoan Passage (and its adjacent passages). The volume transport for each pathway is also determined by counting the number of particles on the pathway. The volume transport thus calculated at a certain location is different from the actual volume transport at that location, and their ratio indicates the percentage contribution of the water coming through the Samoan Passage to the total volume transport.

It should be noted that each particle in the current method does not correspond to a water mass in the conventional sense as it does not experience mixing of water (or transformation of the water mass). Each particle does not have information about dilution, but dispersion of particles represents mixing. A significantly large number of particles are released for the sake of interpreting mixing in that way.

## Supplementary Information

Supplementary Information 1.

Supplementary Information 2.

Supplementary Information 3.

## Data Availability

All figures are produced by using the libraries of Python (https://www.python.org/) (e.g., NumPy, https://numpy.org/; Matplotlib, https://matplotlib.org/). All data are available from the authors upon reasonable request.

## References

[1] Broecker WS (1991). The great ocean conveyor. Oceanography.

[2] Schmitz WJ (1995). On the interbasin-scale thermohaline circulation. Rev. Geophys..

[3] Talley LD (2013). Closure of the global overturning circulation through the Indian, Pacific, and Southern Oceans. Oceanogr..

[4] Wijffels SE (1996). The water masses and circulation at 10°N in the Pacific. Deep Sea Res. I.

[5] Lumpkin R, Speer K (2007). Global ocean Meridional overturning. J. Phys. Oceanogr..

[6] Rudnick DL (1997). Direct velocity measurements in the Samoan Passage. J. Geophys. Res..

[7] Kawabe M, Yanagimoto D, Kitagawa S, Kuroda Y (2005). Variations of the deep western boundary current in Wake Island Passage. Deep Sea Res. I.

[8] Voet G (2016). Warming and weakening of the Abyssal flow through Samoan Passage. J. Phys. Oceanogr..

[9] Kawabe M, Fujio S (2010). Pacific ocean circulation based on observation. J. Oceanogr..

[10] van Sebille E (2018). Lagrangian ocean analysis: Fundamentals and practices. Ocean Model.

[11] Shah SHAM, Primeau FW, Deleersnijder E, Heemink AW (2017). Tracing the ventilation pathways of the deep North Pacific Ocean using Lagrangian particles and Eulerian tracers. J. Phys. Oceanogr..

[12] Waterhouse AF (2014). Global patterns of diapycnal mixing from measurements of the turbulent dissipation rate. J. Phys. Oceanogr..

[13] de Lavergne C (2020). A Parameterization of local and remote tidal mixing. J. Adv. Model. Earth Sys..

[14] Munk WH, Wunsch C (1998). Abyssal recipes II: Energetics of tidal and wind mixing. Deep Sea Res..

[15] Furuichi N, Hibiya T, Niwa Y (2008). Model-predicted distribution of wind-induced internal wave energy in the world’s oceans. J. Geophys. Res..

[16] Tsujino H, Hasumi H, Suginohara N (2000). Deep Pacific circulation controlled by vertical diffusivity at the lower thermocline depths. J. Phys. Oceanogr..

[17] Oka A, Niwa Y (2013). Pacific deep circulation and ventilation controlled by tidal mixing away from the sea bottom. Nat. Comm..

[18] Kawasaki T, Hasumi H, Tanaka Y (2021). Role of tide-induced vertical mixing in the deep Pacific Ocean circulation. J. Oceanogr..

[19] de Lavergne C, Madec G, Roquet F, Holmes RM, McDougall TJ (2017). Abyssal ocean overturning shaped by seafloor distribution. Nature.

[20] Woodgate RA, Aagaard K, Weingartner TJ (2005). Monthly temperature, salinity, and transport variability of the Bering Strait through flow. Geophys. Res. Lett..

[21] Matsumoto K (2007). Radiocarbon-based circulation age of the world oceans. J. Geophys. Res..

[22] Speich S (2002). Tasman leakage: A new route in the global ocean conveyor belt. Geophys. Res. Lett..

[23] Garrison, T. *Oceanography: An Invitation to Marine Science* (Brooks/Cole-Thomson Learning, Belmont, Calif., 2005).

[24] Skinner BJ, Porter SC (1995). The Blue Planet.

[25] Nakano H (2021). Effects of eddies on the subduction and movement of water masses reaching the 137°E section using Lagrangian particles in an eddy-resolving OGCM. J. Oceanogr..

[26] Wilson C (2021). Significant variability of structure and predictability of Arctic Ocean surface pathways affects basinwide connectivity. Commun. Earth Environ..

[27] Niwa Y, Hibiya T (2014). Generation of baroclinic tide energy in a global three-dimensional numerical model with different spatial grid resolutions. Ocean Model.

[28] Gargett AE, Holloway G (1984). Dissipation and diffusion by internal wave breaking. J. Mar. Res..

[29] St. Laurent, L. C., Toole, J. M. & Schmitt, R. W. Buoyancy forcing by turbulence above rough topography in the Abyssal Brazil Basin. *J. Phys. Oceanogr.* **31**, 3476–3495 (2001).

[30] Kunze E (2017). Internal-wave-driven mixing: Global geography and budgets. J. Phys. Oceanogr..

[31] Goto Y, Yasuda I, Nagasawa M, Kouketsu S, Nakano T (2021). Estimation of Basin-scale turbulence distribution in the North Pacific Ocean using CTD-attached thermistor measurements. Sci. Rep..

[32] Gent PR, Willebrand J, McDougall TJ, McWilliams JC (1995). Parameterizing eddy-induced tracer transports in ocean circulation models. J. Phys. Oceanogr..

[33] Roemmich D, Hautala S, Rudnick D (1996). Northward abyssal transport through the Samoan passage and adjacent regions. J. Geophys. Res..

