# Supplementary material for: Deep water pathways in the North Pacific Ocean revealed by Lagrangian particle tracking
Source: Sci Rep. 2022 Apr 22;12:6238. doi: 10.1038/s41598-022-10080-8 (PMC9033868; doi:10.1038/s41598-022-10080-8)

Figure S1. The trajectories of particles randomly selected from all. Colors indicate depths. The numbers of total particles (N) and plotted particles (n) are written in the panels. The destinations are (a) the Southern Ocean and (b) the Indian Ocean.


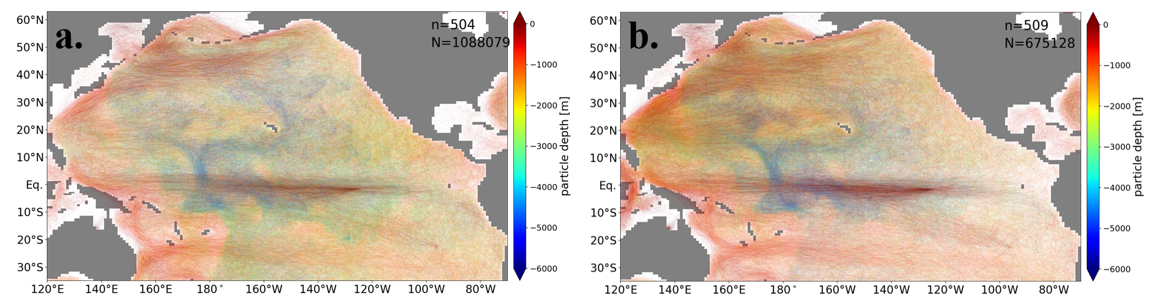

Supplement: Supplementary file 1 — Supplementary Information 1. [file 41598_2022_10080_MOESM1_ESM.docx]
