# Supplementary material for: Deep water pathways in the North Pacific Ocean revealed by Lagrangian particle tracking
Source: Sci Rep. 2022 Apr 22;12:6238. doi: 10.1038/s41598-022-10080-8 (PMC9033868; doi:10.1038/s41598-022-10080-8)
Supplement: Supplementary file 3 — Supplementary Information 3. [file 41598_2022_10080_MOESM3_ESM.docx]

**Table S1.** Dependency of horizontal diffusivity on mean residence time of particles for each destination. Horizontal diffusivity of 1000 m^2^ s^-1^ is employed for the “standard” experiments. The result is obtained from the tracking for 3000 years.

| Destination | Horizontal diffusivity (m^2^ s^-1^) | | |
| --- | --- | --- | --- |
|  | 500 | 1000 | 2000 |
| Southern Ocean | 610 | 533 | 431 |
| Indian Ocean | 804 | 672 | 537 |
| Arctic Ocean | 878 | 728 | 584 |
| Atmosphere | 955 | 804 | 647 |
